# Supplementary material for: Divergent self-association properties of paralogous proteins TRIM2 and TRIM3 regulate their E3 ligase activity
Source: Nat Commun. 2022 Dec 8;13:7583. doi: 10.1038/s41467-022-35300-7 (PMC9732051; doi:10.1038/s41467-022-35300-7)
Supplement: Supplementary file 1 — Supplementary Information [file 41467_2022_35300_MOESM1_ESM.pdf]

Supplementary Information for:

Divergent self-association properties of paralogous proteins TRIM2 and TRIM3 regulate their E3 ligase activity

Diego Esposito[1], Jane Dudley-Fraser[1], Acely Garza-Garcia[2], and Katrin Rittinger[1]*

[1]Molecular Structure of Cell Signalling Laboratory, The Francis Crick Institute, 1 Midland Road, London, NW1 1AT, United Kingdom

[2]Mycobacterial Metabolism and Antibiotic Research Laboratory, The Francis Crick Institute, 1 Midland Road, London, NW1 1AT, United Kingdom

*Correspondence: katrin.rittinger@crick.ac.uk

Contents:

Supplementary Table 1

Supplementary Figures 1 - 7

Supplementary Table 1: SAXS parameters and structure statistics for TRIM2 and TRIM3 refinement.

**Data collection**	**TRIM2 RING**	**TRIM3 RING**
Beamline	SWING at Soleil	
q range ($Å^{-1}$)	0.0082–0.66	
Detector	EigerX4M in vacuum	
Column	Bio-SEC 3 Agilent 100 Å	
Flow rate (ml/min)	0.2	
Sample volume (µl)	100	
Sample concentration (mg/ml)	10	
Temperature (°C)	15	
**Structural parameters**		
**Reciprocal Space**		
Rg (Å) Guinier	19.6 ± 0.1	19.0 ± 0.2
I(0) (cm^{-1})	0.02000 ± 0.00002	0.02300 ± 0.00001
qRg limit	1.29	1.04
**Real Space**		
Rg (Å) P(R)	19.7 ± 0.04	19.5 ± 0.03
I(0) (cm^{-1})	0.02000 ± 0.00002	0.02280 ± 0.00002
Rc (Å)	14.0	9.3
Dmax (Å)	66	74
Porod volume ($Å^3$)	29905	18610
**Molecular mass determination**		
Theoretical MW (kDa)	10.76	10.72
DATPOROD MW (kDa) (Vp/1.7)	17.6	10.9
SAXS MoW2 (q = 0.25 $Å^{-1}$)	19.0	10.4

**TRIM3 RING *ab-initio* and structural modelling**	
**Dammif**	
Number of calculated envelopes	25
Number of final accepted envelopes	24
Normalized Spatial Discrepancy	0.7 ± 0.1
χ^2 (all)	1.80 ± 0.04
χ^2 (best)	1.74
**Xplor-NIH**	
Number of calculated structure	100
Size of final ensemble	10
C^{α} RMSD (N-terminal aa. 2-18) (Å)	11 ± 5
χ^2 (all)	1.34 ± 0.04
χ^2 (best)	1.29

**Data analysis software**	
Primary Data Reduction	Foxtrot
Data processing	Primus & Scatter
Ab-initio modelling	Dammif
Structural modelling	Xplor-NIH
Computation of model intensities	FoXS
3D graphics representation	Pymol

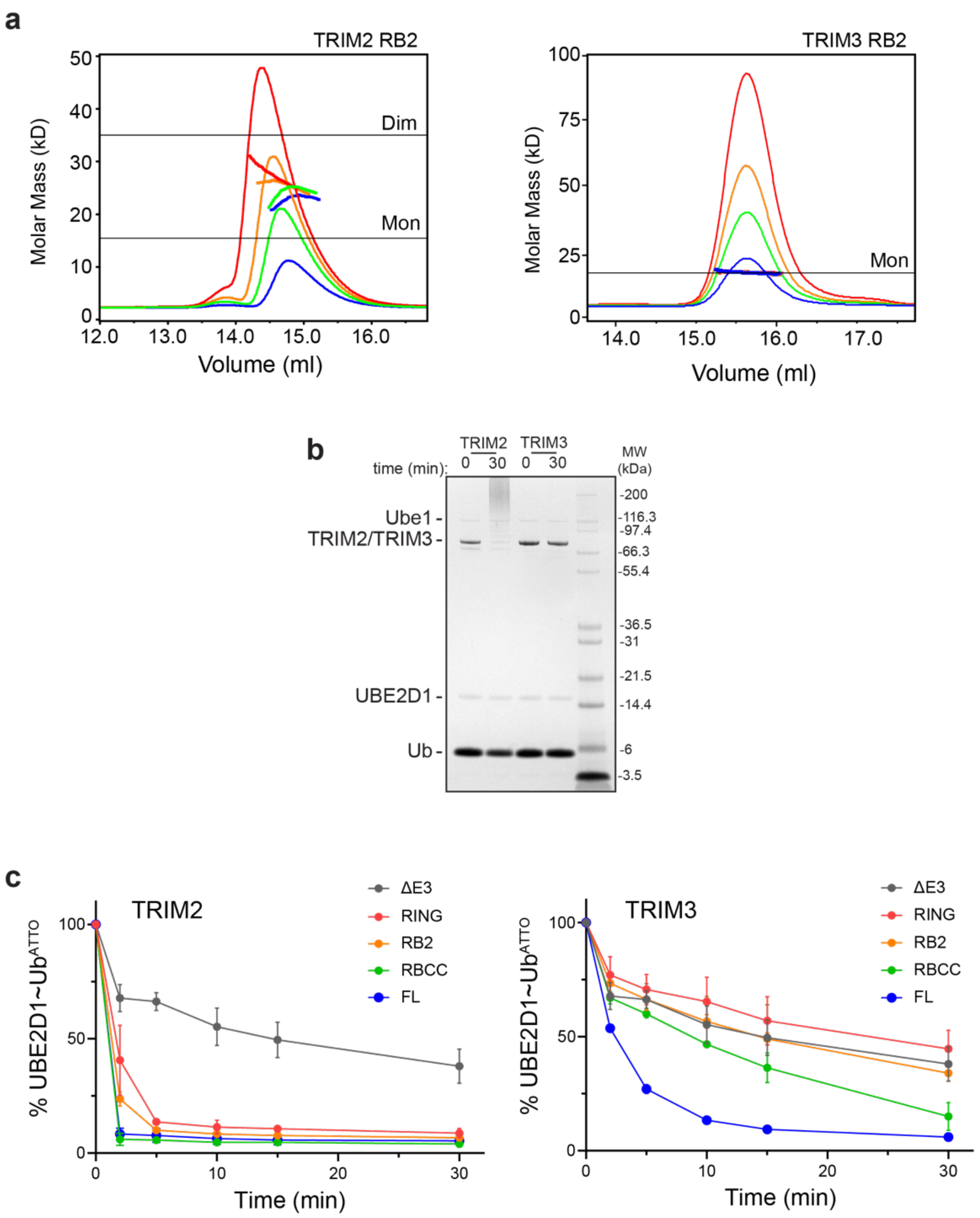

Supplementary Figure 1. Oligomeric state and catalytic activity of TRIM2 and TRIM3.

(a) SEC-MALLS of RB2 constructs of TRIM2 and TRIM3. The proteins were analyzed at a concentration of 5 (red), 3 (orange), 2 (green) and 1 mg/ml (blue). (b) Coomassie-stained SDS-gel to detect auto-ubiquitination of full length TRIM2 and TRIM3 after 30 minutes at 4 μM concentration with 0.5 μM E1, 2.5 μM UBE2D1 and 50 μM ubiquitin. The Mark12^{TM} was used as molecular weight marker. Auto-ubiquitination assays with full length TRIM2 and TRIM3 have been done at least twice ($n \geq 2$). (c) Quantification of the UBE2D1~Ub^{ATTO} discharge

assay where the loss of the E2-Ub^{ATTO} is plotted *vs* reaction time. The data are presented as the mean value ± SD of three independent experiments (n = 3) for the TRIM2 and TRIM3 fragments and full length proteins and of four independent experiments (n = 4) in the absence of E3. Source data is provided as a Source Data file.

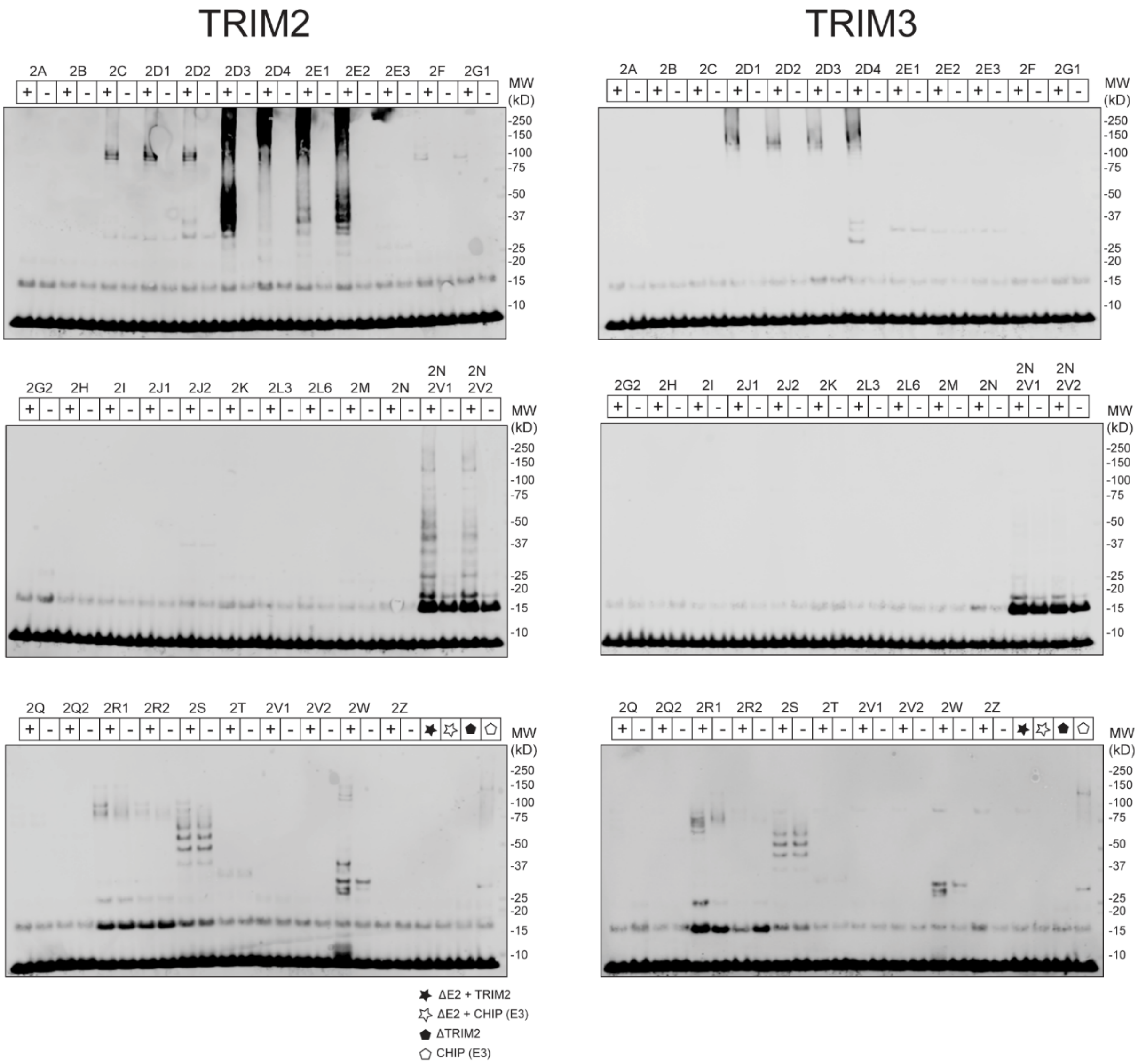

Supplementary Figure 2. E2^{scan} experiments.

E2^{scan} (version 2, Ubiquigent) experiments showing the activity of full length TRIM2 and TRIM3 with different E2 conjugating enzymes. The reactions were run concomitantly in the presence (+) and absence (-) of E3. Control experiments with no E2 (ΔE2) and with CHIP E3 ligase were used to validate the assay. The E2^{scan} experiments were performed once (n = 1). Source data is provided as a Source Data file.

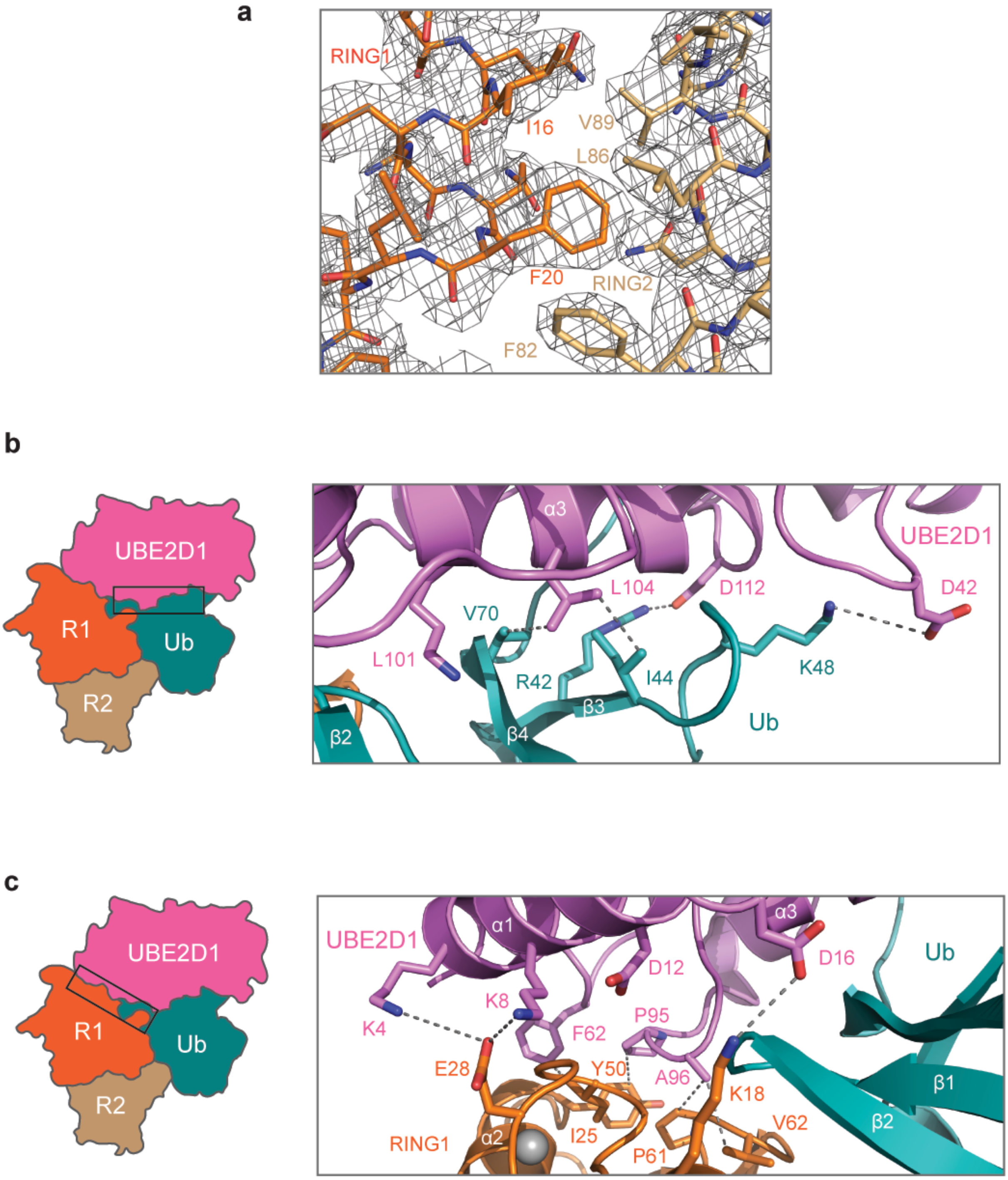

Supplementary Figure 3. Close-ups of the E2/Ub and E2/TRIM2 RING interfaces.

(a) 2Fo-Fc electron density map (grey) contoured at 1σ of a region of the RING1-RING2 interface. (b) Graphical illustration and close-up cartoon representations of the residues involved in the interfaces between UBE2D1 and the conjugated ubiquitin molecule (c) and the

TRIM2 RING1 protomer in the crystal structure of the TRIM2 RING/UBE2D1~Ub complex. The illustration excludes the equivalent E2~Ub conjugate bound to RING2.

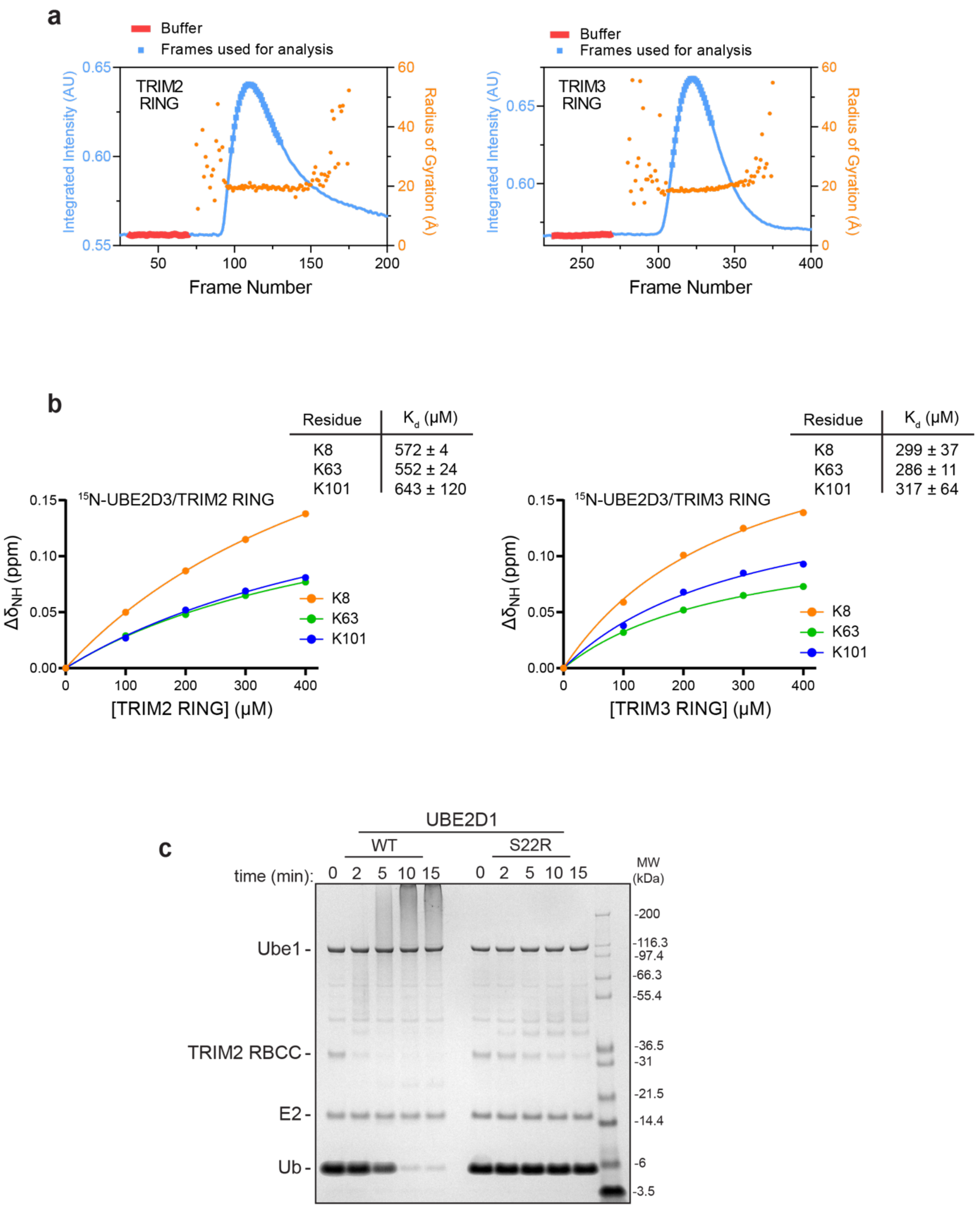
a
Buffer
Frames used for analysis
TRIM2 RING
TRIM3 RING
Integrated Intensity (AU)
Radius of Gyration (Å)
Frame Number
b
Residue
K$_d$ (µM)
K8 572 ± 4
K63 552 ± 24
K101 643 ± 120
K8 299 ± 37
K63 286 ± 11
K101 317 ± 64
^{15}N-UBE2D3/TRIM2 RING
^{15}N-UBE2D3/TRIM3 RING
$\Delta\delta_{NH}$ (ppm)
[TRIM2 RING] (µM)
[TRIM3 RING] (µM)
c
UBE2D1
WT
S22R
time (min): 0 2 5 10 15 0 2 5 10 15
MW (kDa)
Ube1
TRIM2 RBCC
E2
Ub
-200
-116.3
-97.4
-66.3
-55.4
-36.5
-31
-21.5
-14.4
-6
-3.5

Supplementary Figure 4. SEC-SAXS analysis, NMR spectroscopy and effect of UBE2D1 backside S22R mutation.

(a) SEC-SAXS data: integrated intensity of recorded small angle X-ray scattering as a function of frames recorded off the Bio SEC-3 100 Å Agilent column at 0.2 ml/min flow rate. Plotted as orange dots are the values of the derived radii of gyration for the background subtracted recorded profiles. Highlighted are the frames used for the analysis. (b) Fitting of residues K8, K63 and K101 backbone amide proton and nitrogen nuclei chemical shift perturbations in the ^{1}H-^{15}N HSQC spectrum of ^{15}N-labelled UBE2D3 as a function of the concentration of added TRIM2 (left) and TRIM3 (right) RING domain with a 1:1 ligand:protein binding model (E2 + RING ↔ E2/RING). (c) Coomassie-stained SDS gel of an auto-ubiquitination assay of the RBCC of TRIM2 with UBE2D1 wild type and S22R backside mutant. The assay was performed with TRIM2 RBCC at 4 µM concentration with 1 µM E1, 2 µM UBE2D1 and 50 µM ubiquitin. The Mark12™ was used as molecular weight marker. The TRIM2 tripartite motif auto-ubiquitination experiment with the UBE2D1 carrying the S22R mutation was performed in one replicate (n = 1). The rate of auto-ubiquitination of TRIM2 is greatly reduced when the allosteric ubiquitin binding site on the E2 is impaired. Source data is provided as a Source Data file.

a

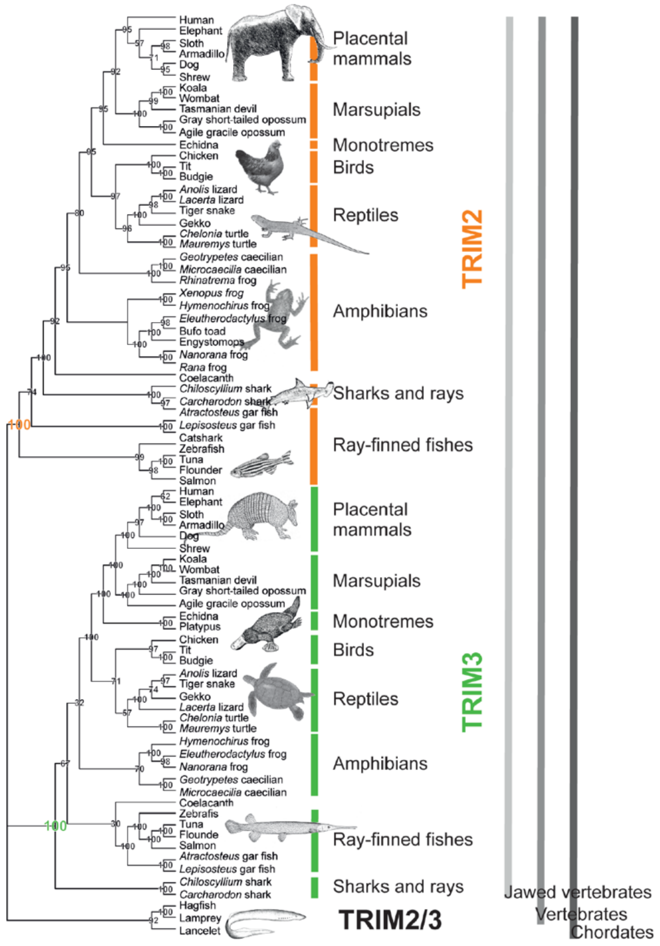
Placental mammals
Marsupials
Monotremes
Birds
Reptiles
Amphibians
Sharks and rays
Ray-finned fishes
TRIM2
TRIM3
TRIM2/3
Jawed vertebrates
Vertebrates
Chordates

b

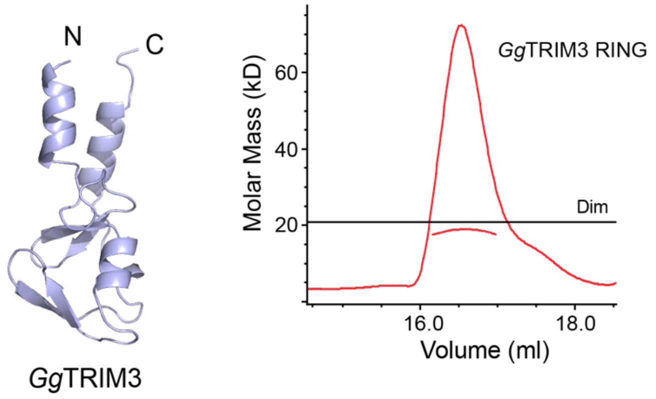
N
C
GgTRIM3
GgTRIM3 RING
Molar Mass (kD)
Volume (ml)
Dim
60
40
20
16.0
18.0

Supplementary Figure 5. Phylogenetic tree of TRIM2 and TRIM3 and oligomeric state of GgTRIM3 RING.

(a) Maximum-likelihood phylogenetic tree of TRIM2 and TRIM3 from selected vertebrate species spanning all extant vertebrate classes. The tree suggests that TRIM2 and TRIM3 are paralogous genes that arose in the first common ancestor of jawed vertebrates. Hagfish and lampreys, the only extant vertebrates without a jaw, as well as basal chordates such as lancelets, have a single TRIM2/3 gene. The tree was calculated with IQ-Tree using the coding DNA sequences of the full-length genes. Branch support values are shown as percentages and were calculated using 1000 ultrafast bootstrap replicates. (b) *G.gallus* TRIM3 RING AlphaFold2 structure model and SEC-MALLS of the RING domain of *G.gallus* TRIM3. The protein was analyzed at a concentration of 5 mg/ml. Source data is provided as a Source Data file.

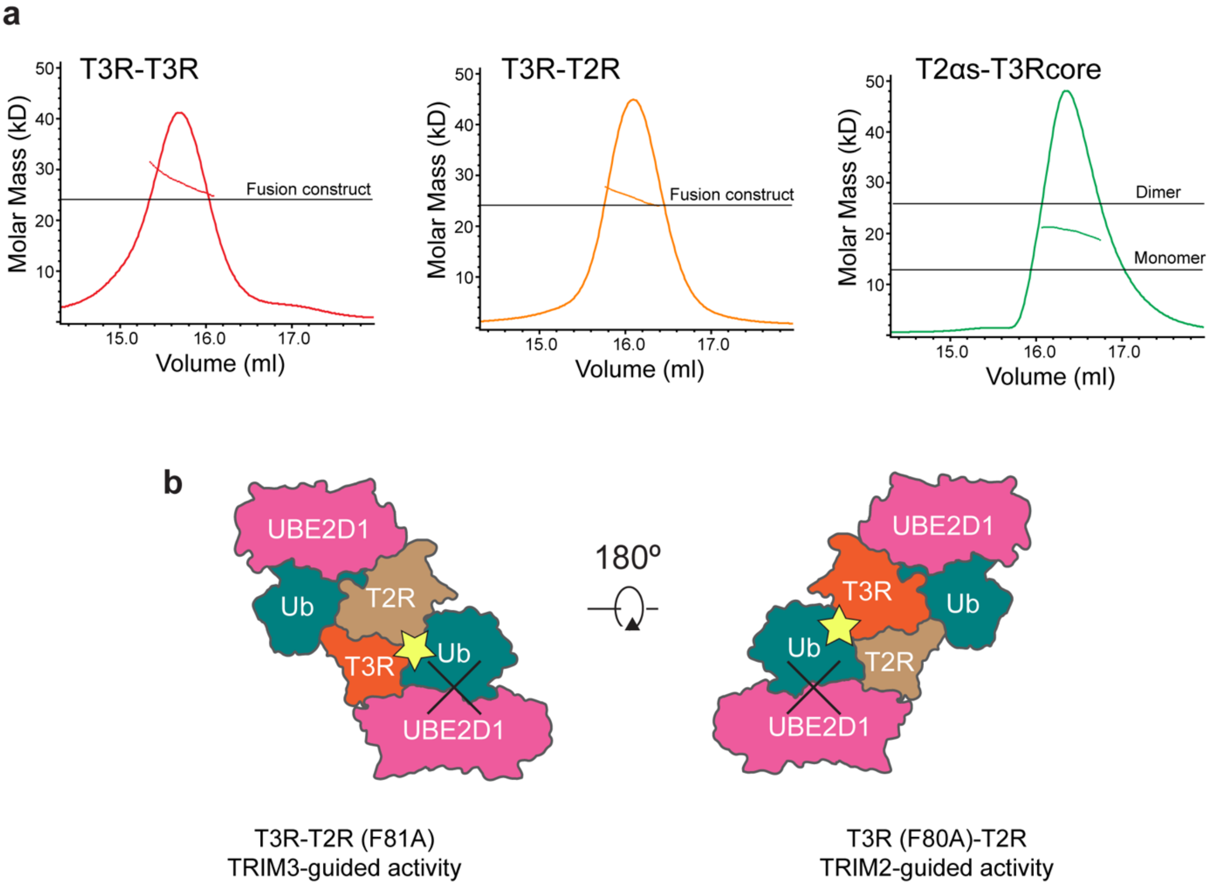

Supplementary Figure 6. TRIM2 and TRIM3 fusion and chimera constructs.

(a) SEC-MALLS of TRIM2/TRIM3 fusion and chimera constructs. Molecular mass of constructs: T3R-T3R 23.6 kDa, T3R-T2R 23.6 kDa, T2αs-T3Rcore 13.0 kDa. Proteins were analyzed at a concentration of 5 mg/ml, the highest concentration used in the experiments with other TRIM2 and TRIM3 constructs. (b) Graphical illustration of the RING/UBE2D1~Ub complex based on our crystal structure adapted to the T3R-T2R fusion where one of the TRIM2 RING domains is substituted by TRIM3. The stars highlight the sites of the F81A mutation in TRIM2 and equivalent position (F80A) in TRIM3 RING. The mutations impair the ability of the respective RING to stabilize the E2~Ub closed conformation (highlighted with a cross) and therefore the observed activity is guided by the active adjacent RING protomer. Source data is provided as a Source Data file.

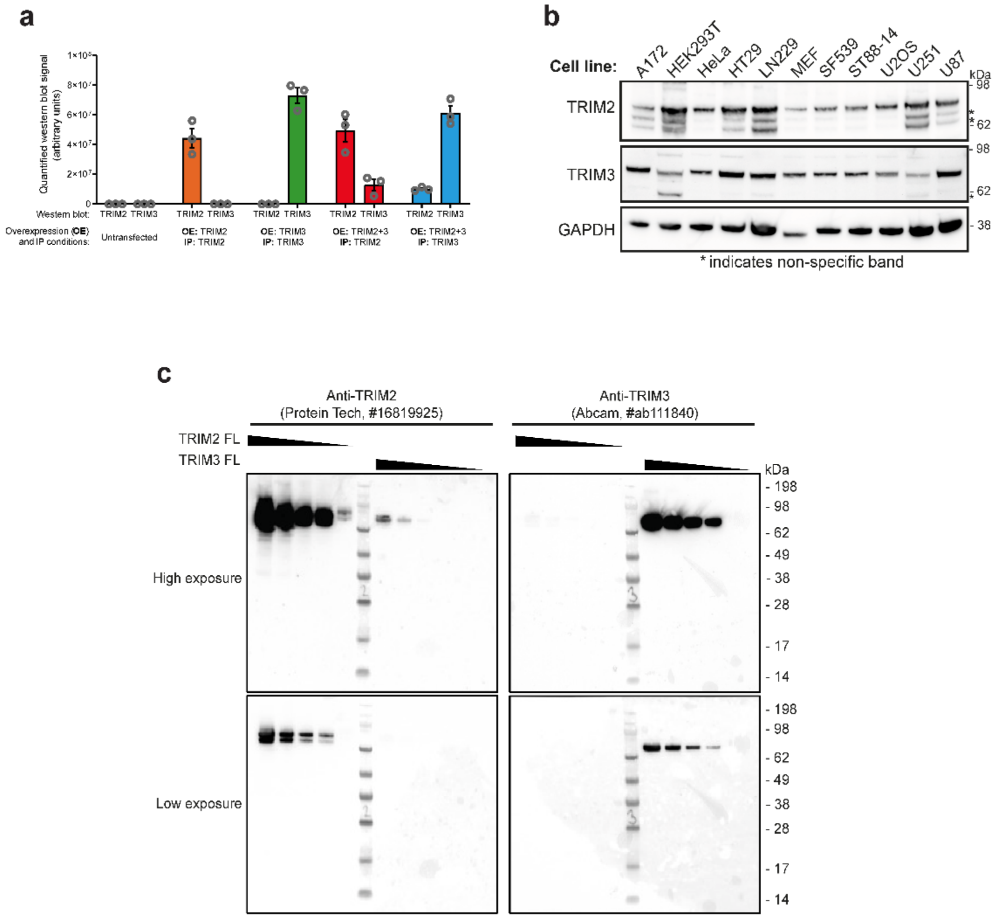

Supplementary Figure 7. Quantification of co-immunoprecipitation from mammalian cells and validation of cell lines and antibodies for use in TRIM2 and TRIM3 cellular interaction studies.

(a) Quantification of GFP-TRIM2 or FLAG-TRIM3 signal from western blotting analysis of experiment described in Figure 6a where proteins are overexpressed (OE) in HEK293T cells and isolated by immunoprecipitation (IP) using their respective tags. Co-immunoprecipitation suggests the proteins interact in cells. The values for each TRIM protein are reported as mean ± SD of three independent duplicates shown as grey circles. (b) Western blotting demonstrating TRIM2 and TRIM3 expression levels in different mammalian cell lines. (c) Validation of TRIM2 and TRIM3 antibody specificity using 1, 0.5, 0.1, 0.05, and 0.01 μg of

recombinant full-length TRIM2 and TRIM3, representing one repetition. Source data is provided as a Source Data file.
