# Supplementary material for: Divergent self-association properties of paralogous proteins TRIM2 and TRIM3 regulate their E3 ligase activity
Source: Nat Commun. 2022 Dec 8;13:7583. doi: 10.1038/s41467-022-35300-7 (PMC9732051; doi:10.1038/s41467-022-35300-7)
Supplement: Supplementary file 3 — Reporting summary [file 41467_2022_35300_MOESM3_ESM.pdf]

nature portfolio

Corresponding author(s): Katrin Rittinger

Last updated by author(s): Nov 23, 2022

Reporting Summary

Nature Portfolio wishes to improve the reproducibility of the work that we publish. This form provides structure for consistency and transparency in reporting. For further information on Nature Portfolio policies, see our Editorial Policies and the Editorial Policy Checklist.

Statistics

For all statistical analyses, confirm that the following items are present in the figure legend, table legend, main text, or Methods section.

n/a	Confirmed	
☐	☒	The exact sample size (n) for each experimental group/condition, given as a discrete number and unit of measurement
☐	☒	A statement on whether measurements were taken from distinct samples or whether the same sample was measured repeatedly
☐	☒	The statistical test(s) used AND whether they are one- or two-sided<br>*Only common tests should be described solely by name; describe more complex techniques in the Methods section.*
☒	☐	A description of all covariates tested
☒	☐	A description of any assumptions or corrections, such as tests of normality and adjustment for multiple comparisons
☐	☒	A full description of the statistical parameters including central tendency (e.g. means) or other basic estimates (e.g. regression coefficient) AND variation (e.g. standard deviation) or associated estimates of uncertainty (e.g. confidence intervals)
☐	☒	For null hypothesis testing, the test statistic (e.g. F, t, r) with confidence intervals, effect sizes, degrees of freedom and P value noted<br>*Give P values as exact values whenever suitable.*
☒	☐	For Bayesian analysis, information on the choice of priors and Markov chain Monte Carlo settings
☒	☐	For hierarchical and complex designs, identification of the appropriate level for tests and full reporting of outcomes
☒	☐	Estimates of effect sizes (e.g. Cohen's d, Pearson's r), indicating how they were calculated

Our web collection on statistics for biologists contains articles on many of the points above.

Software and code

Policy information about availability of computer code

Data collection	Xray diffraction data were collected on beamline IO3 at the Diamond Light Source (Oxford, UK). SAXS data were collected at the SWING beamline at SOLEIL (GIF-sur-YVETTE CEDEX, France). Other data were collected on Odyssey CLx imaging system (LI-COR), DAWN-HELEOS-II laser photometer (Wyatt Technology), Differential refractometer (Optilab TrEX), Bruker 800MHz NMR spectrometer.
Data analysis	Dials (v2020.03.1), Aimless (v6.2), Coot (v0.9.6), Phenix (Phaser.MR and Phenix.Refine) (v1.20.1), CCP4 (refmac) (v7.1.018), Pymol (v2.5.1), ImageStudio Lite (v5.2), ImageLab (v6.1.0), GraphPad (v9), ATSAS (v3.0), SCATTER (v3.0), FoxS (https://modbase.compbio.ucsf.edu/foxs/), Xplor-NIH (v2.48), CCPNMR (v2.4.2), IQ-Tree (v1.6.11), FoXS (https://modbase.compbio.ucsf.edu/foxs/), AlphaFold2 (https://github.com/deepmind/alphafold, The Francis Crick Institute local installation).

For manuscripts utilizing custom algorithms or software that are central to the research but not yet described in published literature, software must be made available to editors and reviewers. We strongly encourage code deposition in a community repository (e.g. GitHub). See the Nature Portfolio guidelines for submitting code & software for further information.

Data

Policy information about availability of data

All manuscripts must include a data availability statement. This statement should provide the following information, where applicable:

- Accession codes, unique identifiers, or web links for publicly available datasets
- A description of any restrictions on data availability
- For clinical datasets or third party data, please ensure that the statement adheres to our policy

The atomic coordinates and the structure factors have been deposited to the Protein Data Bank with the accession code 7ZJ3. Other Protein Data Bank accession codes used in this study: 5FEY (TRIM32 RING); 5FER (TRIM25-RING/UBE2D1~Ub); 6YXE (TRIM69); 4TKP (TRIM5α/UBE2N) and 5EGG (UBE2D3). All the data that

support the conclusions in this study are available with the paper and the source data files are provided with the article. All other relevant data are available from the corresponding author upon reasonable request.

Field-specific reporting

Please select the one below that is the best fit for your research. If you are not sure, read the appropriate sections before making your selection.

- [x] Life sciences
- [ ] Behavioural & social sciences
- [ ] Ecological, evolutionary & environmental sciences

For a reference copy of the document with all sections, see nature.com/documents/nr-reporting-summary-flat.pdf

Life sciences study design

All studies must disclose on these points even when the disclosure is negative.

Sample size	No statistical method was used to predetermine the sample size. X-ray data were obtained from a single crystal to satisfactory redundancy/completeness. In vitro assays were replicated a number of times according to a procedure described in the Methods section. All experiments were executed using sample sizes based on well established protocols in the field. To ensure appropriate data reproducibility at least two, but mostly three or four replicates were executed during experiments. The exact number of replicates for each experiment is reported in the corresponding Figure legend and the data are provided in the Source Data file.
Data exclusions	X-ray reflections were truncated at CC1/2<0.5. No other data were excluded.
Replication	All assays were repeated multiple independent times with the number of replicates indicated in each figure legend where data were quantified. A detailed description of the assays is reported in the Methods section of the manuscript.
Randomization	Randomization was not executed as the data did not require this type of statistical approach. Experiments performed in this study are independent replicates and therefore randomization and covariates was not applicable in our case.
Blinding	Blinding was not necessary as for the experiments described in the manuscript scientist bias could not have any impact on the data or the final measurement.

Reporting for specific materials, systems and methods

We require information from authors about some types of materials, experimental systems and methods used in many studies. Here, indicate whether each material, system or method listed is relevant to your study. If you are not sure if a list item applies to your research, read the appropriate section before selecting a response.

Materials & experimental systems

n/a	Involved in the study	
[ ]	[x]	Antibodies
[ ]	[x]	Eukaryotic cell lines
[x]	[ ]	Palaeontology and archaeology
[x]	[ ]	Animals and other organisms
[x]	[ ]	Human research participants
[x]	[ ]	Clinical data
[x]	[ ]	Dual use research of concern

Methods

n/a	Involved in the study	
[x]	[ ]	ChIP-seq
[x]	[ ]	Flow cytometry
[x]	[ ]	MRI-based neuroimaging

Antibodies

Antibodies used	Mouse anti-GFP (Roche, #11814460001, clones 7.1 and 13.1, 1:1,000), Mouse anti-FLAG (Merck, #A8592, clone M2, 1:10,000), Mouse anti-Ubiquitin (Invitrogen, #13-1600, clone Ubi-1, 1:1,000), Mouse anti-TRIM2 (Protein Tech, #16819925/67342-1-IG-l50UL, clone 1H9C2, 1:500), Rabbit anti-TRIM3 (Abcam, #ab111840, polyclonal, 1:500), Mouse anti-GAPDH (Millipore, #MAB374, clone 6C5, 1:2,000), Goat anti-rabbit-HRP (Dako, #P0399, polyclonal,1:2,000), Goat anti-mouseHRP (Dako, #P0447, polyclonal, 1:2,000), Goat anti-mouse IgG IRDye 800CW (LICOR, #926-32210, polyclonal, 1:8,000), Goat anti-mouse-Alexa594 (ThermoFisher, #A11032, polyclonal, 1:200), Goat anti-rabbit-Alexa488 (ThermoFisher, #A11008, polyclonal, 1:200).
Validation	All antibodies used for this study have been validated and the relative information can be found on the manufacturer websites as listed below.<br>Mouse anti-GFP: https://www.sigmaaldrich.com/GB/en/product/roche/11814460001<br>Mouse anti-FLAG: https://www.sigmaaldrich.com/GB/en/product/sigma/f3165?gclid=CjwKCAjwsJ6TBhAIEiwAfl4TWOsd18kdyBPX3RxEqNJ_9vto5-0Vg_-Tgi08PW9hLJAggP4NLsY3DRoCMmYQAvD_BwE<br>Mouse anti-Ubiquitin Ubi-1: https://www.thermofisher.com/antibody/product/Ubiquitin-Antibody-clone-Ubi-1-Monoclonal/13-1600<br>Mouse anti-TRIM2: https://www.ptglab.com/Products/Pictures/pdf/67342-1-Ig.pdf<br>Rabbit anti-TRIM3: https://www.abcam.com/TRIM3-antibody-ab111840.html

Mouse anti-GAPDH: https://www.merckmillipore.com/GB/en/product/Anti-Glyceraldehyde-3-Phosphate-Dehydrogenase-Antibody-clone-6C5,MM_NF-MAB374?ReferrerURL=https%3A%2F%2Fwww.ecosia.org%2F&bd=1
Goat anti-rabbit-HRP: https://www.citeab.com/antibodies/3288354-p0399-swine-anti-rabbit-immunoglobulins-hrp-affinit
Goat anti-mouse-HRP: https://www.citeab.com/antibodies/3288336-p0447-goat-anti-mouse-immunoglobulins-hrp-affinity
Goat anti-mouse IgG IRDye 800CW: https://www.licor.com/bio/reagents/irdye-800cw-goat-anti-mouse-igg-secondary-antibody
Goat anti-mouse-Alexa594: https://www.thermofisher.com/antibody/product/Goat-anti-Mouse-IgG-H-L-Highly-Cross-Adsorbed-Secondary-Antibody-Polyclonal/A-11032
Goat anti-rabbit-Alexa488: https://www.thermofisher.com/antibody/product/Goat-anti-Rabbit-IgG-H-L-Cross-Adsorbed-Secondary-Antibody-Polyclonal/A-11008

Eukaryotic cell lines

Policy information about cell lines

Cell line source(s)	HEK293T cells (original origin: female human embryo), HeLa (original origin: female adult human cervical cancer), MEF (original origin: mouse embryo), HT29 (original origin: female adult human colorectal cancer), U2OS (original origin: female adult human osteosarcoma), LN229 (original origin: female adult human glioblastoma), SF539 (original origin: female adult human glioma), U87 (original origin: male adult human glioma), U251 (original origin: adult glioblastoma), A172 (original origin: male adult human glioblastoma), ST88-14 (original origin: male adult human peripheral nerve sheath cancer), and Sf9 (clonal isolate of Spodoptera frugiperda Sf21 cells (IPLB-Sf21-AE)) were obtained from the Francis Crick Institute Cell Services STP.
Authentication	Cells were authenticated by STR profiling by Francis Crick Institute Cell Services STP.
Mycoplasma contamination	All cells were negative fro mycoplasma contaminaton tested by fluorescence staining, agar culture, and PCR testing by Francis Crick Institute Cell Services STP.
Commonly misidentified lines (See ICLAC register)	We have used no commonly misidentified cell lines.
